# SARS-CoV-2 infection triggers widespread host mRNA decay leading to an mRNA export block

**DOI:** 10.1261/rna.078923.121

**Published:** 2021-11

**Authors:** James M. Burke, Laura A. St Clair, Rushika Perera, Roy Parker

**Affiliations:** 1 Department of Biochemistry, University of Colorado Boulder, Boulder, Colorado 80303, USA; 2 Center for Vector-Borne and Infectious Diseases, Department of Microbiology, Immunology and Pathology, Colorado State University, Fort Collins, Colorado 80523, USA; 3 Center for Metabolism of Infectious Diseases, Colorado State University, Fort Collins, Colorado 80523, USA; 4 Howard Hughes Medical Institute, University of Colorado Boulder, Boulder, Colorado 80303, USA; 5 BioFrontiers Institute, University of Colorado Boulder, Boulder, Colorado 80303, USA

**Keywords:** SARS-CoV-2, interferon, RNase L, IRF3, mRNA export, mRNA decay, innate immunity

## Abstract

The transcriptional induction of *interferon* (*IFN*) genes is a key feature of the mammalian antiviral response that limits viral replication and dissemination. A hallmark of severe COVID-19 disease caused by SARS-CoV-2 is the low presence of IFN proteins in patient serum despite elevated levels of *IFN*-encoding mRNAs, indicative of post-transcriptional inhibition of IFN protein production. Here, we performed single-molecule RNA visualization to examine the expression and localization of host mRNAs during SARS-CoV-2 infection. Our data show that the biogenesis of type I and type III *IFN* mRNAs is inhibited at multiple steps during SARS-CoV-2 infection. First, translocation of the interferon regulatory factor 3 (IRF3) transcription factor to the nucleus is limited in response to SARS-CoV-2, indicating that SARS-CoV-2 inhibits RLR-MAVS signaling and thus weakens transcriptional induction of *IFN* genes. Second, we observed that *IFN* mRNAs primarily localize to the site of transcription in most SARS-CoV-2 infected cells, suggesting that SARS-CoV-2 either inhibits the release of *IFN* mRNAs from their sites of transcription and/or triggers decay of *IFN* mRNAs in the nucleus upon exiting the site of transcription. Lastly, nuclear-cytoplasmic transport of *IFN* mRNAs is inhibited during SARS-CoV-2 infection, which we propose is a consequence of widespread degradation of host cytoplasmic basal mRNAs in the early stages of SARS-CoV-2 replication by the SARS-CoV-2 Nsp1 protein, as well as the host antiviral endoribonuclease, RNase L. Importantly, *IFN* mRNAs can escape SARS-CoV-2-mediated degradation if they reach the cytoplasm, making rescue of mRNA export a viable means for promoting the immune response to SARS-CoV-2.

## INTRODUCTION

The severe acute respiratory syndrome coronavirus 2 (SARS-CoV-2) is the cause of the COVID-19 pandemic. Since SARS-CoV-2 will remain endemic in human populations (Lavine et al. 2021), development of COVID-19 treatments is paramount. Several clinical trials are currently underway that modulate the innate immune response to treat COVID-19, including treatment with interferon (IFN) proteins (NCT04350671; NCT04388709; CT04647695; NCT04552379). However, the innate immune response to SARS-CoV-2 infection is not well-understood.

During the innate immune response to viral infection, the detection of viral double-stranded RNA (dsRNA) results in the transcriptional induction of mRNAs encoding for cytokines, including type I and type III IFNs, which are exported to the cytoplasm for translation (Jensen and Thomsen 2012; Ivashkiv and Donlin 2014; Lazear et al. 2019). The induction of *interferon* mRNAs occurs through the binding of dsRNA to Rig-I-like receptors (RLR), leading to the formation of a MAVS signaling complex, which then activates multiple kinases that phosphorylate IRF3, a transcription factor that translocates to the nucleus to activate transcription of the interferon genes (collectively referred to as RLR-MAVS-IRF3 signaling) (for review, see Rehwinkel and Gack 2020). Interferon proteins are secreted from infected cells and prime an antiviral state in both infected and noninfected cells via autocrine and paracrine signaling. This limits viral replication capacity and promotes the function of innate and adaptive immune cells at sites of infection, which reduces viral loads and limits viral dissemination to secondary sites of infection.

Despite the potent antiviral activities of IFNs, it is currently controversial whether IFNs promote COVID-19 disease via their proinflammatory functions, or whether the low production of IFNs in response to SARS-CoV-2 contributes to COVID-19 disease progression. In support of the former, *IFN*-encoding mRNAs are elevated in patients with severe COVID-19 symptoms (Lee et al. 2020; Wilk et al. 2020; Zhou et al. 2020). In support of the latter, IFN proteins are relatively low in patients with severe COVID-19 symptoms (Blanco-Melo et al. 2020; Hadjadj et al. 2020). While seemingly contradictory, these findings are nonetheless consistent with observations that SARS-CoV-2 induces, yet antagonizes, IFN protein production (Lei et al. 2020; Li et al. 2021).

How IFNs are inhibited during SARS-CoV-2 infection is unclear. Several SARS-CoV-2 proteins, including Nsp1, have been proposed to antagonize the RLR-MAVS-IRF3-mediated induction of IFNs (Lei et al. 2020; Xia et al. 2020). In addition, two studies have suggested that host mRNA export is inhibited during SARS-CoV-2 infection, which might also reduce the induction of interferon proteins (Finkel et al. 2021; Zhang et al. 2021). The mechanism by which SARS-CoV-2 inhibits mRNA export has been suggested to be through direct interactions of the viral Nsp1 with the host nuclear export factor 1 (NXF1) (Zhang et al. 2021).

We hypothesized that SARS-CoV-2 might actually inhibit mRNA export by triggering the widespread degradation of host mRNAs. This possibility is suggested by the activation of oligo(A) synthetases (OAS) in response to viral dsRNA that produce 2′–5′ oligo(A), thereby activating RNase L (Chakrabarti et al. 2011). RNase L triggers widespread decay of host mRNAs (Burke et al. 2019; Rath et al. 2019), which is known to lead to inhibition of mRNA export (Burke et al. 2021). Moreover, SARS-CoV-2 infection is known to trigger RNase L activation (Li et al. 2021). To test this possibility, we used single-cell and single-molecule imaging of host and viral mRNAs to address how SARS-CoV-2 affects the stability of host mRNAs as well as the induction and export of interferon mRNAs. We observed that both activation of the host antiviral endoribonuclease, RNase L, and expression of SARS-CoV-2 Nsp1 leads to rapid and near-complete decay of host basal mRNAs prior to the induction of *IFN* genes, resulting in the inhibition of nuclear-cytoplasmic transport of *IFN* mRNAs. In addition, we demonstrate that SARS-CoV-2 limits the biogenesis of *IFN* mRNAs by reducing their transcription via attenuation of IRF3 nuclear localization, inhibiting their release from their sites of transcription, and/or triggering their nuclear degradation. These findings define new mechanisms by which *IFN* mRNA biogenesis is perturbed during SARS-CoV-2 infection that have implications for transcriptomic analyses of SARS-CoV-2 infection, IFN-based treatments, the development of drugs to inhibit SARS-CoV-2-Nsp1-mediated mRNA decay, and the use of drugs that regulate nuclear import of proteins and nuclear export of mRNAs to regulate the innate immune response to SARS-CoV-2.

## RESULTS

### Generation of WT and RNase L-KO A549 cells conducive to SARS-CoV-2 infection

**FIGURE 1.**
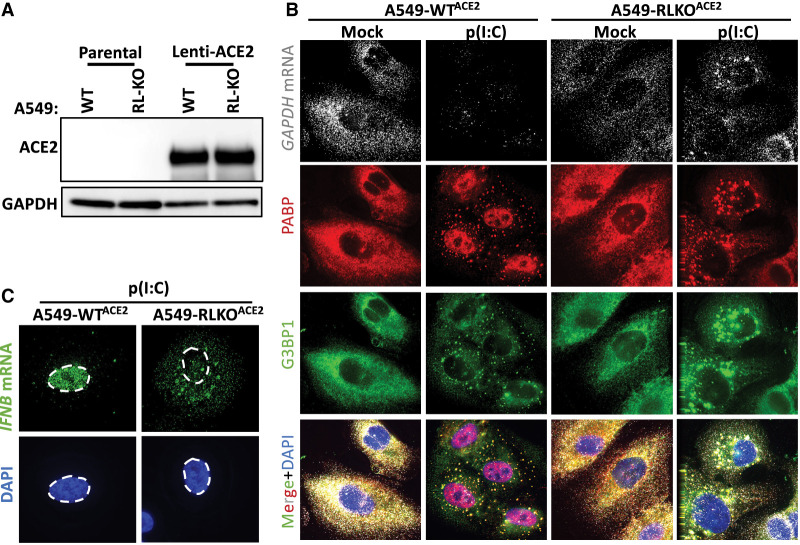
Generation and characterization of WT and RNase L-KO A549 cells that express ACE2. (*A*) Immunoblot analysis to confirm ACE2 expression in parental (WT) and RNase L-KO (RL-KO) A549 cells. (*B*) Single-molecule fluorescence in situ hybridization (smFISH) for GAPDH mRNA and immunofluorescent assay for RNA-binding proteins PABP and G3BP1 that enrich in RNase L-dependent bodies (RLBs) in WT cells and stress granules in RL-KO cells 4 h post-lipofection of poly(I:C). (*C*) smFISH for *IFNB* mRNA in WT^ACE2^ and RL-KO^ACE2^ cells 4 h post-lipofection of poly(I:C).

To study SARS-CoV-2 infections in a cell line with a robust innate immune response we transduced parental (WT) and RNase L knockout (RL-KO) A549 lung carcinoma cell lines (Burke et al. 2019) with an ACE2-encoding lentivirus to make them permissive to SARS-CoV-2 infection. We confirmed ACE2 expression by western blot analysis (Fig. 1A). We also confirmed several RNase L-dependent phenotypes in response to poly(I:C) lipofection in WT^ACE2^ but not RL-KO^ACE2^ cells (Burke et al. 2019, 2020, 2021), including degradation of *GAPDH* mRNA, the generation of small stress granule-like foci (RNase L-dependent bodies; RLBs), inhibition of stress granule assembly, PABP translocation to the nucleus, and nuclear retention of *IFNB* mRNA (Fig. 1B,C). This demonstrates that these A549 cells expressing the ACE2 receptor have a normal innate immune response to dsRNA.

### smFISH analysis of SARS-CoV-2 mRNAs

**FIGURE 2.**
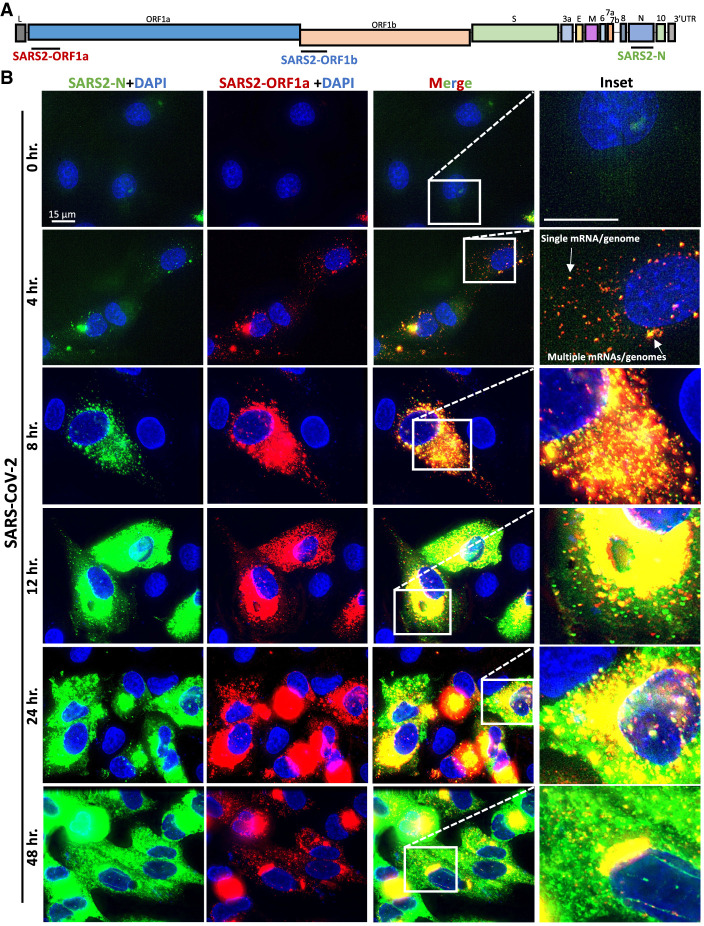
Single-molecule analysis of SARS-CoV-2 genomic and subgenomic RNAs. (*A*) Schematic to show the location of smFISH probe sets targeting the different regions of SARS-CoV-2 mRNA. The ORF1a and ORF1b target the full-length genome, whereas the N probes target both the full-length genome and subgenomic RNAs. (*B*) smFISH for SARS-CoV-2 full length genome (ORF1a probes) and subgenomic RNAs (N probes) at indicated times post-infection with SARS-CoV-2 (MOI = 5).

To identify SARS-CoV-2-infected cells, we generated single-molecule fluorescence in situ hybridization (smFISH) probe sets that target the ORF1a, ORF1b, or N regions of the SARS-CoV-2 genomic mRNA (Fig. 2A). The ORF1a and ORF1b probes would be expected to detect the full-length (FL) genome, whereas the N probes would detect both the FL-genome and subgenomic (SG) mRNAs (Fig. 2A).

We costained A549-WT^ACE2^ cells at multiple times post-infection (MOI = 5) with ORF1a and N smFISH probes. By 4 h post-infection, we observed small and dispersed foci (∼100 copies/cell) that costained for ORF1a and N RNA, which we suggest are individual SARS-CoV-2 genomes/full-length mRNAs (Fig. 2B; Supplemental Fig. S1A). In addition, we observed larger structures that contain multiple genomes, which are likely replication factories (RFs) and/or concentrated sites of translation or mRNA processing. At 8 h post-infection, SARS-CoV-2 genome copies increased ∼10-fold (to ∼1000 copies/cell) and subgenomic RNAs became abundant throughout the cell (Fig. 2B; Supplemental Fig. S1AB). From twelve through 48 h post-infection, large RFs concentrated with full-length genome (fluorescent intensity was generally saturating) localized to the perinuclear region of the cell (Fig. 2B). At these later times, subgenomic RNAs (N probes) were more abundant, as these N-positive RNAs only partially localized to the RFs and were mostly dispersed throughout the cytoplasm (Fig. 2B; Supplemental Fig. S1B).

### SARS-CoV-2 infection triggers widespread degradation of host mRNAs independent of RNase L

**FIGURE 3.**
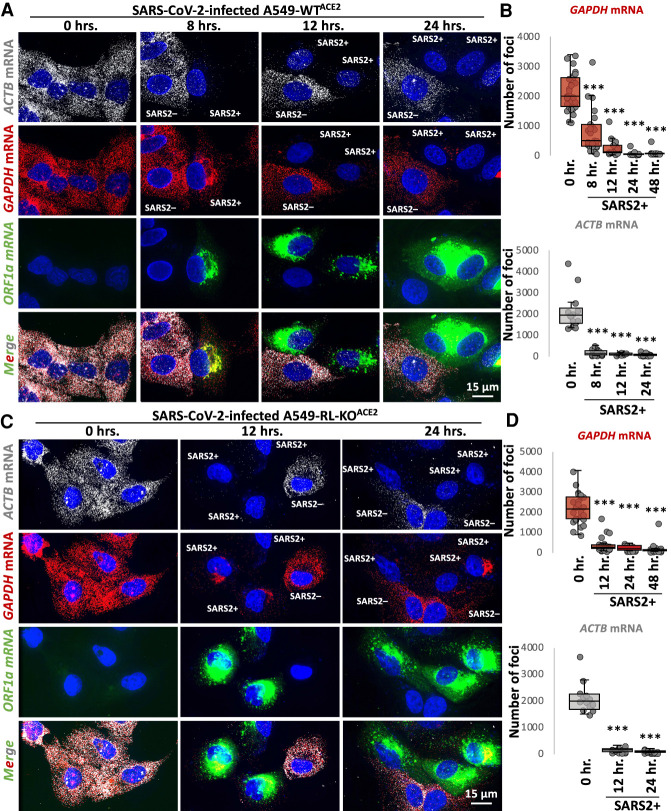
Host mRNA levels rapidly reduce following SARS-CoV-2 infection, independently of RNase L. (*A*) smFISH for host *GAPDH* and *ACTB* mRNAs and SARS-CoV-2 full-length genome (ORF1b) at indicated times post-infection with SARS-CoV-2 (MOI = 5) in WT^ACE2^ A549 cells. (*B*) Graphs show quantification of *GAPDH* and *ACTB* mRNAs as represented in *above* images. (*C*,*D*) Similar to *A* and *B* but in RL-KO^ACE2^ A549 cells. Dots represent individual cells. Between 16 to 30 cells were analyzed per group. Statistical significance ([*] *P* < 0.05; [**] *P* < 0.005; [***] *P* < 0.0005) was determined by *t*-test.

To examine if SARS-CoV-2 infection activated RNase L-mediated mRNA decay, we stained for host *GAPDH* and *ACTB* basal mRNAs by smFISH. We observed a substantial reduction (greater than 10-fold) in *GAPDH* and *ACTB* mRNAs in SARS-CoV-2-infected cells WT^ACE2^ cells as early as 8 h post-infection (Fig. 3A,B). This coincides with early stages of viral replication when viral genomes substantially increased in level from 4 to 8 h post-infection (Fig. 2). Thus, SARS-CoV-2 infection leads to widespread loss of cytosolic host mRNAs.

Unexpectedly, we also observed a reduction in *GAPDH* and *ACTB* mRNAs in SARS-CoV-2-infected RL-KO^ACE2^ cells (Fig. 3C,D). This indicates that the reduction in host basal mRNAs in response to SARS-CoV-2 infection can occur independently of RNase L.

However, several observations indicate that RNase L is activated by SARS-CoV-2 infection. First and consistent with RNase L reducing SARS-CoV-2 replication by approximately fourfold (Li et al. 2021), we observed that RNase L reduced both FL-genome and N-RNA by approximately threefold as compared to the RL-KO^ACE2^ cells (Supplemental Fig. S2A–C). Second, we observed robust RNase L-dependent accumulation of PABP in the nucleus by 24 h post-infection (Supplemental Fig. S2D), which is a previously reported consequence of RNase L activation (Burke et al. 2019). In contrast, despite widespread mRNA degradation in the RL-KO^ACE2^ cells, PABP did not translocate to the nucleus (Supplemental Fig. S2D). Lastly, we observed small punctate PABP-positive foci consistent with RLBs (RNase L-dependent bodies) in WT^ACE2^ but not RLKO^ACE2^ cells (Supplemental Fig. S2E; Burke et al. 2019, 2020). We note that we did not observe G3BP1/PABP-positive stress granules in RL-KO^ACE2^ cells (Supplemental Fig. S2D), likely due to the loss of host mRNAs required for stress granule assembly.

Combined, these data indicate that SARS-CoV-2 infection leads to widespread degradation of host mRNAs both by the activation of RNase L, and by a second RNase L-independent mechanism.

### SARS-CoV-2 Nsp1 expression is sufficient for degradation of host basal mRNAs

The degradation of host basal mRNAs in the RL-KO^ACE2^ cells could either be an RNase L-independent host response or mediated by viral proteins. The Nsp1 protein encoded by SARS-CoV-1 is known to reduce host mRNA levels during coronavirus infection (Kamitani et al. 2006), possibly by inhibiting translation and promoting mRNA decay (Narayanan et al. 2008; Schubert et al. 2020). Nsp15 is an endoribonuclease that processes viral RNA (Bhardwaj et al. 2008), but could potentially cleave host mRNAs. Therefore, we tested whether the Nsp1 or Nsp15 proteins encoded by SARS-CoV-2 could be responsible for host mRNA decay.

**FIGURE 4.**
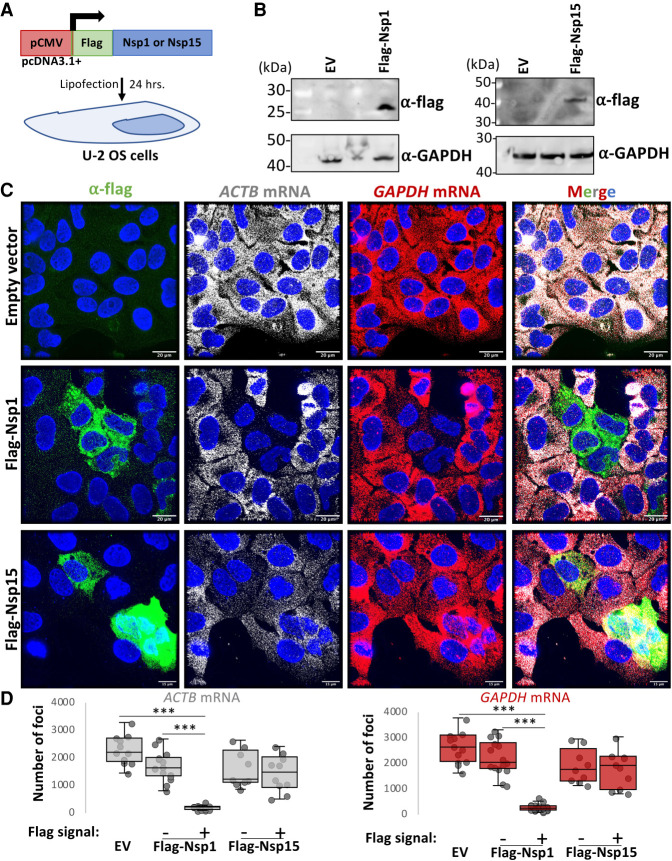
SARS-CoV-2 Nsp1 expression is sufficient for degradation of host basal mRNAs. (*A*) Schematic of Flag-tagged SARS-CoV-2 protein expression vector transfected into U2-OS cells. (*B*) Immunoblot for Flag confirmed expression of Flag-tagged Nsp1 and Nsp15 expression at expected size (Nsp1 ∼20 kDa; Nsp15 ∼40 kDa) in cells transfected with respective expression vectors but not empty vector (EV). Note, the unlabeled lanes between the EV and Nsp1/Nsp15 vectors are plasmid clones that did not express the proteins and were not used for subsequent experiments. (*C*) Immunofluorescence assay for Flag and smFISH for *ACTB* and *GAPDH* mRNAs in U-2 OS cells 24 h post-transfection with either pcDNA3.1+ (empty vector; EV), Flag-Nsp1, or Flag-Nsp15 expression vectors. (*D*) Quantification of *ACTB* and *GAPDH* mRNAs as represented in *C*. Dots represent individual cells. Between 10–22 cells analyzed per group. Statistical significance ([*] *P* < 0.05; [**] *P* < 0.005; [***] *P* < 0.0005) was determined by *t*-test.

We generated Flag-tagged SARS-CoV-2 Nsp1 or Nsp15 expression vectors and transfected them into U-2 OS cells (Fig. 4A,B). In cells transfected with Flag-Nsp1 (identified by staining for the Flag epitope), both *ACTB* and *GADPH* mRNAs were strongly reduced in comparison to cells that did not stain for Flag or cells transfected with empty vector (Fig. 4C,D). Expression of Flag-Nsp15 did not result in notable reduction of *ACTB* and *GADPH* mRNAs (Fig. 4C,D). These data indicate that expression of SARS-CoV-2 Nsp1 protein is sufficient to initiate the widespread degradation of host basal mRNAs, arguing that Nsp1 contributes to host mRNA degradation during SARS-CoV-2 infection.

### Alterations to type I and type III *IFN* mRNA biogenesis during SARS-CoV-2 infection

The observations that SARS-CoV-2 both activates RNase L (Supplemental Fig. S2) and promotes decay of host basal mRNAs via Nsp1 (Figs. 3, 4) suggests that *IFN* mRNAs might be retained in the nucleus due to an mRNA export block triggered by widespread cytosolic RNA degradation (Burke et al. 2021). Given this possibility, we examined the expression of *IFN* mRNAs by smFISH during SARS-CoV-2 infection. These experiments revealed three important insights into how SARS-CoV-2 affects IFN production.

#### SARS-CoV-2 infection triggers IFN gene induction

**FIGURE 5.**
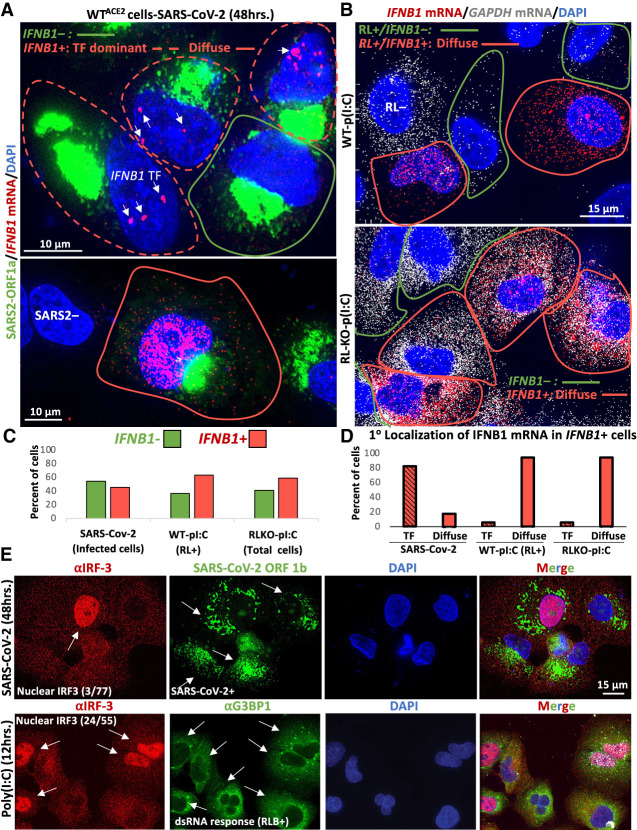
*IFN* mRNAs are retained at the site of transcription during SARS-CoV-2 infection. (*A*) smFISH for *IFNB1* mRNA and SARS-CoV-2 ORF1a 48 h post-infection. Two fields of view are shown. In the *top* image, the cell boundary of SARS-CoV-2-positive cells that stain for *IFNB1* are demarcated by red line, whereas IFNB1 mRNA-negative cells are demarcated by green line. Cells that contain *IFNB1* transcription site foci (TF) but lack abundant disseminated *IFNB1* mRNA are demarcated by dashed red line. The *lower* image shows a SARS-CoV-2-infected cell that contains abundant and diffuse *IFNB1* mRNA in the nucleus and cytoplasm. Cells that do not stain for SARS-CoV-2 are labeled SARS2-. (*B*) smFISH for *IFNB1* mRNA and *GAPDH* mRNA 16 h post-poly(I:C) transfection in WT and RL-KO A549 cells. In WT cells, 12% do not activate RNase L (RL−). Of the 88% of cells that activate RNase L, 63% (55% of total cells) also induce abundant and disseminated *IFNB1* mRNA (red line), whereas 37% of RL+ cells do not induce IFNB1 (green line). Fifty-nine percent of RL-KO cells induce abundant disseminated IFNB1 mRNA (red line), whereas 41% do not (green line). (*C*) Histograms quantifying the percent of SARS-CoV-2 infected cells, poly(I:C)-transfected WT cells that activate RNase L (GAPDH mRNA-negative cells), and poly(I:C)-transfected RL-KO cells that induce *IFNB1*, as represented in *A* and *B*. (*D*) Histograms quantifying the percent of *IFNB1*-positive cells in which IFNB1 smFISH staining is predominantly localized to *IFNB1* transcription site foci (TF) or diffuse. (*E*) Immunofluorescence assay for IRF3 translocation from the cytoplasm to the nucleus in response to either SARS-CoV-2 infection (48 h p.i.; MOI = 5) or poly(I:C) lipofection (12 h). The fraction of cells displaying robust nuclear IRF3 staining is shown in the IRF3 images. For SARS-CoV-2 infection, smFISH for SARS-CoV-2 ORF-1b was used to identify infected cells indicated by arrows. For cells undergoing dsRNA response to poly(I:C), G3BP1 immunofluorescence was used to identify RNase L-dependent bodies (RLBs), as indicated by white arrows.

We observed that SARS-CoV-2 infection often triggers the transcriptional induction of *IFN* genes. This is based on the observation that 45% of SARS-CoV-2-infected A549-WT^ACE2^ cells stain positive for abundant disseminated *IFNB1* mRNA and/or nascent *IFNB1* transcripts at *IFNB1* genomic loci (Fig. 5A–C), referred to as transcription site foci (TF) (Burke et al. 2019). The lack of *IFNB1* induction in 55% of SARS-CoV-2-infected cells is likely, and in part, due to the inherent heterogeneity of the innate immune response in A549 cells (Burke et al. 2019), since 37% of A549-WT cells that were transfected with poly(I:C) (as determined by RNase L activation) did not induce *IFNB1* expression (Fig. 5B,C).

#### SARS-CoV-2 limits IFN induction and biogenesis and/or causes nuclear degradation of IFN mRNAs

The above data indicate that SARS-CoV-2 replication triggers RLR-MAVS-IRF3 signaling. However, several observations suggest that SARS-CoV-2 disrupts *IFN* mRNA biogenesis (Fig. 5A–E). Specifically, of the SARS-CoV-2-infected cells that induced *IFNB1*, greater than 82% contained *IFNB1* transcription site foci but lacked abundant diffuse *IFNB1* mRNAs (Fig. 5A,D). In these cells (termed “TF dominant”), *IFNB1* mRNAs were few in number (<50 foci) and limited to the vicinity of the *IFNB1* transcription site foci (Fig. 5A; top image). Less than 18% of SARS-CoV-2 infected cells displayed abundant *IFNB1* mRNAs that had disseminated away from the *IFNB1* site of transcription (Fig. 5A; lower image, 5D). We observed a similar effect staining for *IFNL1* mRNA (Supplemental Fig. S3A).

Our data indicate that the inability of *IFNB1* mRNA to disseminate away from *IFNB1* transcriptional foci during SARS-CoV-2 infection is not typical of *IFNB1* induction nor a consequence of RNase L activation. Specifically, of the WT or RL-KO A549 cells that induce *IFNB1* in response to poly(I:C) lipofection, greater than 94% displayed widespread dissemination of *IFNB1* mRNA in the nucleus and/or in the cytoplasm, with very few cells (<6%) displaying identifiable *IFNB1* transcriptional foci but lacking disseminated *IFNB1* mRNA (Fig. 5B,D; Supplemental Fig. S3B). Since most WT cells activated RNase L in response to poly(I:C), and all WT cells that induced *IFNB1* also activated RNase L (Fig. 5B,C), RNase L activation does not cause retention of *IFNB1* mRNA at the *IFNB1* transcription site. Further supporting that RNase L does not cause this effect, we observed this phenomenon in SARS-CoV-2-infected RL-KO^ACE2^ cells (Supplemental Fig. S3B).

The TF-dominant phenotype indicates that SARS-CoV-2 possibly interferes with the transcription of *IFN* genes. Consistent with this notion, immunofluorescence assay for IRF3, which shuttles from the cytoplasm to the nucleus upon RLR-MAVS-dependent phosphorylation to promote transcription of *IFN* genes, revealed that only a small fraction (∼4%) of SARS-CoV-2-infected cells displayed robust nuclear IRF3 staining (Fig. 5E). This is in contrast to poly(I:C) lipofection, which causes robust nuclear IRF3 localization that occurs in approximately half of cells that initiated a dsRNA response (as determined by RLB assembly) (Fig. 5E). We interpret this data to mean that SARS-CoV-2 attenuates RLR-MAVS-IRF3 signaling, consistent with previous reports (Lei et al. 2020), thereby resulting in weak accumulation of IRF3 in the nucleus. We suggest that this attenuates *IFNB1* transcriptional output, contributing to the TF-dominant phenotype that most SARS-CoV-2-infected cells display. However, it is possible that alterations in RNA processing, which could prevent the release of mRNAs from transcription sites (Hilleren et al. 2001), and/or that degradation of *IFN* mRNAs upon leaving the transcription site could account for the TF-dominant phenotype during SARS-CoV-2 infection.

#### SARS-CoV-2 infection blocks nuclear export of IFN mRNAs

**FIGURE 6.**
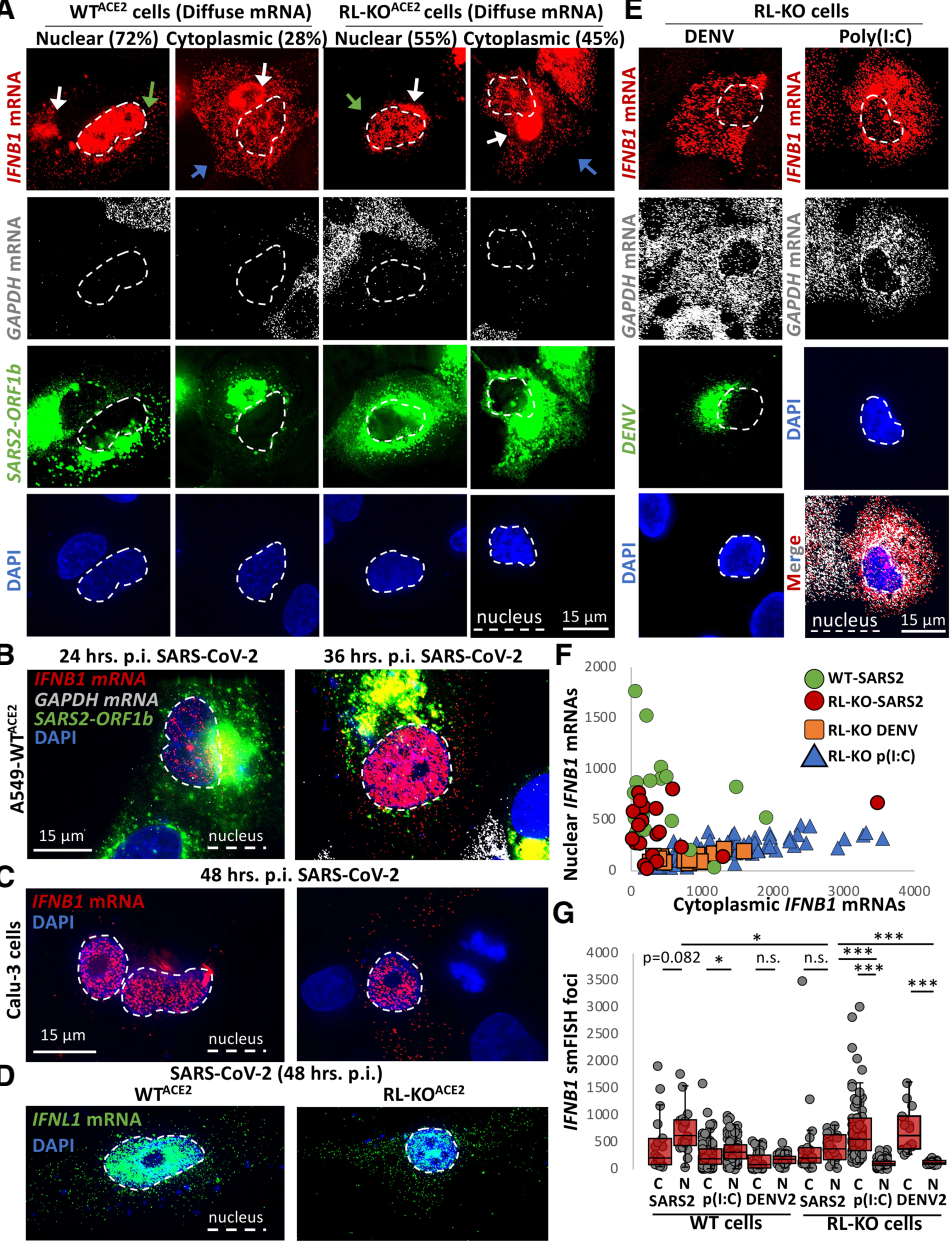
Nuclear-cytoplasmic transport of *IFN* mRNAs is inhibited during SARS-CoV-2 infection. (*A*) smFISH for *IFNB1* mRNA, *GAPDH* mRNA, and SARS-CoV-2 *ORF1b* mRNA in WT^ACE2^ and RL-KO^ACE2^ cells 48 h post-infection with SARS-CoV-2 (MOI = 5). Spectral crossover from the ORF1b-staining of the SARS-CoV-2 RF into the *IFNB1* mRNA channel is indicated by white arrows. The green arrows indicate cells in which *IFNB* mRNA is retained in the nucleus. The blue arrows indicate cells in which *IFNB* mRNA is localized to the cytoplasm. (*B*) Similar to *A* but smFISH was performed at 24- and 36-h post-infection. (*C*) smFISH for *IFNB1* mRNA in Calu-3 cells 48 h post-infection with SARS-CoV-2 (MOI = 5). Two fields of view are shown. (*D*) smFISH for *IFNL1* mRNA in SARS-CoV-2-infected cells 48 h post-infection (MOI = 5). (*E*) Representative smFISH for *IFNB1* and *GAPDH* mRNAs in RL-KO A549 cells 48 h post-infection with DENV (MOI = 0.1) or 16 h post-transfection with poly(I:C). (*F*) Scatter plot quantifying IFNB1 mRNA in the nucleus (*y*-axis) and in the cytoplasm (*x*-axis) in individual WT^ACE2^ or RL-KO^ACE2^ cells infected with SARS-CoV-2, or RL-KO cells 48 h post-infection with DENV2 or 8 h post-transfection with poly(I:C). (*G*) Quantification of *IFNB1* mRNA via smFISH in the nucleus (N) or cytoplasm (C) of either WT^ACE2^ or RL-KO^ACE2^ cells infected with SARS-CoV-2, and WT or RL-KO cells transfected with poly(I:C) or infected with DENV2 as represented in (*A* and *C*). Poly(I:C) and DENV2 data were obtained from Burke et al. (2021). Dots represent individual cells. Between 20 to 125 cells were analyzed per group. Statistical significance ([*] *P* < 0.05; [**] *P* < 0.005; [***] *P* < 0.0005) was determined by *t*-test.

A second mechanism we observed by which SARS-CoV-2 infection limits IFN protein production is by a block in the transport of *IFN* mRNAs from the nucleus to the cytoplasm (Fig. 6A–F). The critical observation is that in SARS-CoV-2 infected A549-WT^ACE2^ cells that induced *IFNB1* and displayed abundant and dispersed *IFNB1* mRNA by 48 h post-infection, 72% retained the majority (>50%) of *IFNB1* mRNAs in the nucleus (Fig. 6A,F). We observed this effect at earlier times post-infection (24 or 36 h) (Fig. 6B), though very few cells contain *IFNB1* mRNA at or before these times. Thus, the inhibition of mRNA export is not necessarily a result of cytopathic effects observed at late times post-infection. We also observed that *IFNB1* mRNA is predominantly retained in the nucleus of SARS-CoV-2-infected Calu-3 cells (Fig. 6C). Lastly, we observed nuclear retention of *IFNL1* mRNA in SARS-CoV-2 infected cells (Fig. 6D).

The nuclear retention of *IFN* mRNAs during SARS-CoV-2 infection is similar to that observed in response to RNase L activation during poly(I:C) lipofection or dengue virus serotype 2 (DENV2) infection (Burke et al. 2021). However, nuclear retention of *IFN* mRNAs during SARS-CoV-2 infection can occur independently of RNase L activation. The key observation is that we observed nuclear retention of *IFNB1* mRNA in SARS-CoV-2-infected RL-KO^ACE2^ cells (Fig. 6A,F). This is in contrast to poly(I:C) lipofection or DENV2 infection in RL-KO cells, in which *IFNB1* mRNA is predominantly localized to the cytoplasm (Fig. 6E,F; Burke et al. 2021). We note that the number of cells displaying nuclear retention and the magnitude of nuclear retained *IFNB1* mRNA was higher in WT^ACE2^ cells in comparison to RL-KO^ACE2^ cells (Fig. 6A,F,G), indicating RNase L activation in response to SARS-CoV-2 infection increases nuclear mRNA retention.

A notable difference between SARS-CoV-2 infection and either poly(I:C) lipofection or DENV infection in RL-KO cells is that basal mRNAs are only degraded in the context of SARS-CoV-2 infection (Fig. 6A,E). This suggests that SARS-CoV-2-mediated mRNA decay might be responsible for inhibiting the nuclear export of *IFNB1* mRNAs, similar to RNase L-mediated mRNA decay (Burke et al. 2021). To better assess this model, we compared nuclear and cytoplasmic *IFNB1* mRNA levels during SARS-CoV-2 infection, poly(I:C) lipofection, or DENV2 infection in both WT and RL-KO cells.

This analysis supports that SARS-CoV-2-mediated mRNA decay, similar to RNase L-mediated mRNA decay, inhibits IFNB1 mRNA export. Specifically, while median *IFNB1* mRNA levels in the cytoplasm are approximately eightfold higher in the nucleus of wild-type cells as compared to RL-KO cells infected with DENV2 or transfected with poly(I:C), they are equivalent in SARS-CoV-2-infected wild-type and RL-KO cells (Fig. 6G). Moreover, the ratio of nuclear to cytoplasmic *IFNB1* mRNA levels in SARS-CoV-2-infected RL-KO cells is comparable to WT cells infected with SARS-CoV-2, DENV2, or transfected with poly(I:C) (Fig. 6G), which are all conditions in which basal mRNAs are lost. Thus, the high percentage of cells displaying nuclear retention of *IFNB1* mRNA is specific to scenarios in which widespread decay of host mRNA occurs, including RNase L activation (Burke et al. 2021) and SARS-CoV-2 infection (Figs. 3, 4).

#### IFN mRNAs escape SARS-CoV-2-mediated mRNA decay

Interestingly, in a fraction of SARS-CoV-2-infected WT^ACE2^ or RL-KO^ACE2^ cells (28% and 45%, respectively) displaying diffuse *IFNB1* mRNA staining, *IFNB1* mRNA was abundant in the cytoplasm despite robust decay of *GAPDH* mRNA (Fig. 6A). These data indicate that the *IFNB1* mRNA at least partially evades both RNase L- and Nsp1- mediated mRNA decay mechanisms during SARS-CoV-2 infection when the *IFNB1* mRNA is successfully exported to the cytoplasm. This is similar to results seen with activation of RNase L either by poly(I:C) transfection or DENV2 infection (Burke et al. 2019, 2021; Rath et al. 2019).

## DISCUSSION

**FIGURE 7.**
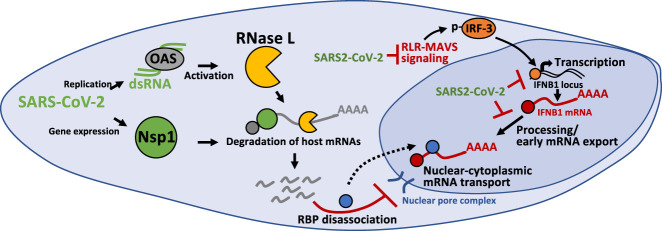
Inhibition of antiviral mRNA biogenesis during SARS-CoV-2 infection. Model of how antiviral mRNA biogenesis is inhibited during SARS-CoV-2 infection. SARS-CoV-2 replication generates double-stranded RNA (dsRNA), which is recognized by OAS and leads to RNase L activation. In addition, SARS-CoV-2 expresses the viral Nsp1 protein. Both RNase L activation and Nsp1 expression result in rapid and widespread decay of host basal mRNAs. We propose that the degradation of host mRNAs results in release of RNA-binding proteins (RBPs), and this perturbs late stages of nuclear-cytoplasmic RNA transport. The sequestration of antiviral mRNAs, such as *IFNB1* mRNA, in the nucleus prevents their association with ribosomes in the cytoplasm, reducing their translation for protein production. In addition, SARS-CoV-2 inhibits the transcription of antiviral genes by reducing nuclear levels of IRF3 via inhibition of RLR-MAVS signaling. Lastly, SARS-CoV-2 alters an aspect of mRNA processing or association with early mRNA export factors, and/or rapidly degrades dsRNA-induced antiviral mRNAs, such as *IFNB1* mRNA. The result of this is the inability of *IFNB1* mRNAs to exit the site of *IFNB1* transcription, preventing their transport to the cytoplasm and reducing their translation.

Several observations support that SARS-CoV-2 infection perturbs *IFN* mRNA biogenesis, limiting *IFN* mRNAs from reaching the cytoplasm where they can be translated (Fig. 7). First, *IFN* genes are induced in ∼45% of SARS-CoV-2-infected cells based on the detection of IFNB mRNAs (Fig. 5A,C,D). This indicates that RLR-MAVS-IRF3/7 signaling is initiated by SARS-CoV-2, consistent with previous reports (Lei et al. 2020; Li et al. 2021). However, we observed that both type I- and type III IFN-encoding mRNAs predominately localize to their sites of transcription in a majority of cells (Fig. 5A,D; Supplemental Fig. S3A), which is atypical of *IFN* induction and not a consequence of RNase L activation (Fig. 5B,D). Our data showing that nuclear localization of IRF3 is low in most SARS-CoV-2 infected cells suggest that SARS-CoV-2 attenuates the RLR-MAVS signaling (Fig. 5E), consistent with previous reports (Lei et al. 2020). Therefore, we suggest that SARS-CoV-2-mediated inhibition of RLR-MAVS signaling reduces nuclear IRF3 levels, which attenuates or interferes with the transcription of IFN genes. In addition, SARS-CoV-2 may also alter some aspect of RNA processing or an early step of mRNA export, either of which is necessary for efficient release of stable mRNAs from transcription sites (Hilleren et al. 2001).

In addition to inhibition of transcriptional induction or early mRNA processing/export, we observed a defect in nuclear-cytoplasmic mRNA transport during SARS-CoV-2 infection. Specifically, we observed that the majority of cells in which IFN mRNAs were released from the sites of transcription retained those *IFN* mRNAs within, but disseminated throughout, the nucleus in SARS-CoV-2-infected cells (Fig. 6A–D). However, the mRNA export block of *IFN* mRNAs appears to be distinct from the accumulation of *IFN* mRNAs at transcription site foci since a similar mRNA export block is triggered by RNase L without reduction of transcription nor trapping of mRNAs at the transcription site in both poly(I:C)-treated and DENV2-infected cells (Fig. 5A–D; Burke et al. 2021). However, we cannot rule out that SARS-CoV-2-mediated inhibition of mRNA export could be mechanistically coupled with the accumulation of *IFN* mRNAs at the site of transcription.

The inhibition of nuclear mRNA export by SARS-CoV-2 infection can be understood as a direct consequence of widespread mRNA degradation in the cytosol. The key observation is that we observed a near-complete loss of host basal mRNAs in response to SARS-CoV-2 within the early replication cycles (4 to 8 h post-infection) (Figs. 2, 3). Since the median half-life of human mRNAs is 10 h (Yang et al. 2003), these data are consistent with accelerated mRNA decay, though our data do not rule out that shut-off of host transcription also contributes to host basal mRNA reduction. The degradation of host mRNAs could be mediated by RNase L activation (Supplemental Fig. S2) and/or the SARS-CoV-2 Nsp1 protein (Fig. 4). Since we have recently shown that RNase L-mediated mRNA decay inhibits mRNA export of *IFN* mRNAs (Burke et al. 2021), these data argue that either RNase L- or SARS-CoV-2-Nsp1-mediated mRNA decay leads to inhibition of host mRNA export. It should be noted that RNase L per se is not required for this export block since we observed *IFN* mRNAs trapped in the nucleus in RNase L knockout cells where widespread mRNA degradation is driven by Nsp1 (Figs. 4, 6A,F,G). Regardless of the nuclease responsible for mRNA destruction, the nuclear retention of *IFN* mRNAs away from ribosomes would consequently reduce IFN protein production in response to SARS-CoV-2 infection.

Although the detailed mechanism of the mRNA export block is unknown, it appears to be a general consequence of any widespread cytosolic mRNA degradation. This mechanism is suggested by the observations that mRNA export blocks occur due to activation of RNase L (Burke et al. 2021), the nuclease SOX produced by Kaposi's sarcoma-associated herpesvirus (KSHV) (Glaunsinger et al. 2005; Kumar and Glaunsinger 2010; Gilbertson et al. 2018), and by degradation of mRNAs by Nsp1 in RL-KO cells (Figs. 3, 4). A likely explanation for the export block is that widespread cytosolic mRNA degradation leads to relocalization of numerous RNA binding proteins to the nucleus (Kumar and Glaunsinger 2010; Gilbertson et al. 2018; Burke et al. 2019; Khong and Parker 2020), which would then compete for the binding of export factors to mRNAs. Consistent with that hypothesis, overexpression of the mRNA export factor NXF1 (Nuclear RNA Export Factor 1) has been suggested to overcome an mRNA export block due to Nsp1 binding to NXF1 (Zhang et al. 2021). However, we anticipate that Nsp1 binding to NXF1 would not be required for inhibition of mRNA export in SARS-CoV-2 infected cells since anytime mRNAs are degraded via RNase L activation, which happens in SARS-CoV-2 infections (Supplemental Fig. S2; Li et al. 2021), there is a robust mRNA export block independent of any viral protein (Burke et al. 2021). Moreover, we observe inhibition of *IFNB1* mRNA export during SARS-CoV-2 infections, which is exported by CRM1-dependent export pathway and is independent of NXF1 (Fig. 6A; Burke et al. 2021). An important issue for future research is to understand the factors that compete for mRNA export once cytosolic mRNAs are degraded.

Despite rapid degradation of host basal mRNAs, SARS-CoV-2 RNAs appeared to be largely unaffected since they increased over time and were only modestly reduced by RNase L (Fig. 2, Supplemental Figs. S1, S2). Similarly, in cells in which *IFNB* mRNAs were exported to the cytoplasm, *IFNB1* mRNAs appeared to be stable since they were abundant despite complete decay of basal mRNAs (Fig. 6A), similar to *IFNB1* mRNA escaping RNase L-mediated mRNA decay (Burke et al. 2019). Importantly, this indicates that *IFN* mRNAs evade SARS-CoV-2-mediated mRNA decay mechanisms, making rescue of host mRNA processing and export a viable option for increasing IFN protein production.

## MATERIALS AND METHODS

### Cell culture

Parental and RNase L-KO (RL-KO) A549, U-2 OS, and HEK293T cell lines are described in Burke et al. (2019). Cells were maintained at 5% CO_2_ and 37°C in Dulbecco's modified Eagle's medium (DMEM) supplemented with fetal bovine serum (FBS; 10% v/v) and penicillin/streptomycin (1% v/v). Cells were routinely tested for mycoplasma contamination by the cell culture core facility. Cells were transfected with poly(I:C) HMW (InvivoGen: tlrl-pic) using 3 µL of lipofectamine 2000 (Thermo Fisher Scientific) per 1 µg or poly(I:C). African green monkey kidney cells (Vero E6, ATCC CRL-1586) were maintained at 5% CO_2_ and 37°C in DMEM supplemented with FBS (10% v/v), 2 mM nonessential amino acids, 2 mM l-glutamine, and 25 mM HEPES buffer. Calu-3 cells (ATCC HTB-55) were maintained at 5% CO_2_ and 37°C in DMEM supplemented with non-heat inactivated FBS (15% v/v), 2 mM nonessential amino acids, 2 mM l-glutamine, and 25 mM HEPES buffer.

### Plasmids

The Flag-Nsp1 and Flag-Nsp15 vectors were generated by ligating a g-block (Integrated DNA Technologies [IDT]) encoding for the Flag and ORF of Nsp1 or Nsp15 between the xho1 and xba1 sites in pcDNA3.1+. Plasmids were sequence verified. The pLEX307-ACE2-puro plasmid was a gift from Alejandro Chavez and Sho Iketani (Addgene plasmid # 158448; http://n2t.net/addgene:158448; RRID:Addgene_158448).

### Viral infections

SARS-CoV-2/WA/20/01 (GenBank MT020880) was acquired from BEI Resources (NR-52881) and used for all infections. The virus was passaged in Vero E6 cells, and viral titer was determined via plaque assay on Vero E6 as previously described in Dulbecco and Vogt (1953). A multiplicity of infection (MOI) of five was used unless otherwise noted. All cell culture and plate preparation work were conducted under biosafety level 2 conditions, while all viral infections were conducted under biosafety level 3 conditions at Colorado State University. For infections, cells were seeded in six-wells format onto coverslips. Twenty-four hours later, cell growth medium was removed, and cells were inoculated with SARS-CoV-2 at the indicated MOI for 1 h at room temperature to allow for viral adherence. After incubation, viral inoculum was removed, cells were washed with 1× PBS, and DMEM supplemented with 2% FBS (v/v) was added to each well. Cells were fixed in 4% paraformaldehyde and phosphate-buffered saline (PBS) for 20 min, followed by three five-minute washes with 1× PBS, and stored in 75% ethanol. Following paraformaldehyde fixation, plates were removed from the BSL3 facility, and stored at 4°C until staining.

### Generation of ACE2-expressing cell lines

HEK293T cells (T-25 flask; 80% confluent) were cotransfected with 2.4 µg of pLenti-pLex307-ACE lentiviral transfer plasmid (Addgene: 158448), 0.8 µg of pVSV-G, 0.8 µg of pRSV-Rev, and 1.4 µg of pMDLg-pRRE using 20 µL of lipofectamine 2000. Media was collected at 24- and 48-h post-transfection and filter-sterilized with a 0.45 µm filter. To generate A549^ACE2^ lines, cells were incubated for 1 h with 1 mL of ACE2-encoding lentivirus stock containing 10 µg of polybrene. Normal medium was then added to the flask and incubated for 24 h. Medium was removed 24 h post-transduction and replaced with selective growth medium containing 2 µg/mL of puromycin (Sigma-Aldrich). Selective medium was changed every 3 d. After 1 wk, selective medium was replaced with normal growth medium. Expression of ACE2 was confirmed via immunoblot analysis (protocol described in Burke et al. 2019) using Anti-Angiotensin Converting Enzyme 2 antibody [EPR4435(2) (Abcam: ab108252)] at 1:1000.

### Single-molecule fluorescent in situ hybridization (smFISH) and immunofluorescence assays

smFISH was performed following manufacturer's protocol (https://biosearchassets.blob.core.windows.net/assets/bti_custom_stellaris_immunofluorescence_seq_protocol.pdf) and as described in Burke et al. (2019) and Burke et al. (2021). GAPDH and ACTB smFISH probes labeled with Quasar 570 Dye (GAPDH: SMF-2026-1) or Quasar 670 Dye (GAPDH: SMF-2019-1) (ACTB: VSMF-2003-5) were purchased from Stellaris. Custom IFNB1, IFNL1, and SARS-CoV-2 smFISH probes (Supplemental Table 1) were designed using Stellaris smFISH probe designer (Biosearch Technologies) available online at http://www.biosearchtech.com/stellaris-designer. Reverse complement DNA oligos were purchased from IDT (Extended data file 1). The probes were labeled with Atto-633 using ddUTP-Atto633 (Axxora: JBS-NU-1619-633), with ATTO-550 using 5-Propargylamino-ddUTP (Axxora; JBS-NU-1619-550), or ATTO-488 using 5-Propargylamino-ddUTP (Axxora; JBS-NU-1619-488) with terminal deoxynucleotidyl transferase (Thermo Fisher Scientific: EP0161) as described in Gaspar et al. (2017).

For immunofluorescence detection, cells were incubated with Rabbit polyclonal anti-PABP antibody (Abcam: ab21060) (1:1000), Mouse monoclonal anti-G3BP antibody (Abcam: ab56574) (1:1000), and IRF-3 (D6I4C) XP Rabbit (Cell Signaling Technologies: mAb #11904) (1:400) primary antibodies for 2 h, washed three times, and then incubated with Goat Anti-Rabbit IgG H&L (Alexa Fluor 647) (Abcam: ab150079) and Goat Anti-Mouse IgG H&L (FITC) (Abcam; ab97022) at 1:2000 for 1 h. After three washes, cells were fixed and then smFISH protocol was performed.

### Microscopy and image analysis

Microscopy was performed as described in Burke et al. (2021). Briefly, coverslips were mounted on slides with VECTASHIELD Antifade Mounting Medium with DAPI (Vector Laboratories; H-1200). Images were obtained using a wide field DeltaVision Elite microscope with a 100× objective using a PCO Edge sCMOS camera. Ten Z planes at 0.2 µm/section were taken for each image. Deconvoluted images were processed using ImageJ with FIJI plugin. *Z* planes were stacked, and minimum and maximum display values were set in ImageJ for each channel to properly view fluorescence. Imaris Image Analysis Software (Bitplane) (University of Colorado-Boulder, BioFrontiers Advanced Light Microscopy Core) was used to quantify smFISH foci in nucleus and cytoplasm. Fluorescent intensity was quantified in ImageJ. Independent replicates were performed to confirm results.

## SUPPLEMENTAL MATERIAL

Supplemental material is available for this article.

## COMPETING INTEREST STATEMENT

Roy Parker is a founder and consultant of Faze Medicines.

## Supplementary Material

Supplemental Material
