# Supplementary material for: SARS-CoV-2 infection triggers widespread host mRNA decay leading to an mRNA export block
Source: RNA. 2021 Nov;27(11):1318–29. doi: 10.1261/rna.078923.121 (PMC8522697; doi:10.1261/rna.078923.121)
Supplement: Supplemental Material [file supp_27_11_1318__DC1.html]

SARS-CoV-2 infection triggers widespread host mRNA decay leading to an mRNA export block — Supplemental Material 

# SARS-CoV-2 infection triggers widespread host mRNA decay leading to an mRNA export block

## Supplemental Material

- Supplemental\_Figures\_S1-S3.docx
- Supplemental\_Table\_1.xlsx
