# Supplementary material for: SARS-CoV-2 infection triggers widespread host mRNA decay leading to an mRNA export block
Source: RNA. 2021 Nov;27(11):1318–29. doi: 10.1261/rna.078923.121 (PMC8522697; doi:10.1261/rna.078923.121)
Supplement: Supplemental Material [file supp_078923.121_Supplemental_Figures_S1-S3.docx]

**Supplemental figures and legends**

**Fig. S1. SARS-CoV-2 genome and mRNA abundance in single-cells at early times post-infection.**

(A) smFISH for ORF1a and N regions of SARS-CoV-2 four- and eight- hours post-infection with SARS-CoV-2. (B) Scatter-plot quantifying smFISH for ORF1a (x-axis) and N region (y-axis), which captures sub-genomic RNAs, at indicated times post-infection. At four hours post-infection, N-targeted RNAs are equivalent to full-length genomes, but are more abundant than full-length genome at eight hours post-infection.

**Fig. S2. RNase L is activated by SARS-CoV-2 infection.**

(A) SARS-CoV-2 full-length genome (ORF1a probes) at indicated times post-infection WT^ACE2^ and RL-KO^ACE2^ cells. (B) Quantification of FL genome (ORF1a) fluorescent intensity in WT^ACE2^ and RL-KO^ACE2^ cells. (C) Quantification of fluorescent intensity of sub-genomic RNA (N probes) in WT^ACE2^ and RL-KO^ACE2^ cells. (D) IFA for stress granule markers PABP and G3BP1 and smFISH for SARS-CoV-2 ORF1a in WT^ACE2^ and RL-KO^ACE2^ cells mock-infected or infected with SARS-CoV-2. (E) IFA for PABP and smFISH for SARS-CoV-2 ORF1a in WT^ACE2^ cells.

**Fig. S3. *IFN* mRNAs primarily localize the site of transcription in SARS-CoV-2-infected cells.**

(A) smFISH for *IFNB1* and *IFNL1* mRNAs in WT^ACE2^ cells forty-eight hours post-infection with SARS-CoV-2. (B) smFISH for *IFNB1* mRNA and SARS-CoV-2 ORF1a forty-eight hours post-infection in RL-KO^ACE2^. SARS-CoV-2-positive cells stain for *IFNB1* (green arrows), whereas others do not (yellow arrow). Cells that contain *IFNB1* transcriptional foci (TF) but lack abundant disseminated *IFNB1* mRNA are indicated by blue arrow
